# Diagnosis of Primary Langerhans Cell Histiocytosis of the Vulva in a Postmenopausal Woman

**DOI:** 10.1155/2013/962670

**Published:** 2013-09-15

**Authors:** Sefa Kurt, Mehmet Tunc Canda, Aycan Kopuz, Dudu Solakoglu Kahraman, Abdullah Tasyurt

**Affiliations:** ^1^Department of Obstetrics and Gynecology, Tepecik Teaching Hospital, Gaziler Caddesi No. 468, Yenisehir, 35110 Izmir, Turkey; ^2^Obstetrics and Gynecology Unit, Kent Hospital, 8229/1 Sokak No. 56, Cigli, 35580 Izmir, Turkey; ^3^Department of Pathology, Tepecik Teaching Hospital, Gaziler Caddesi No. 468, Yenisehir, 35110 Izmir, Turkey

## Abstract

Langerhans cell histiocytosis (LCH) is a very rare disease of female genital tract, most commonly seen in vulva and unusual in postmenopausal period. Herein, we report the 8th case of pure vulvar LCH in a postmenopausal woman. We pay attention to the differential diagnosis in postmenopausal state, features of pathologic diagnosis, and treatment options.

## 1. Introduction

Langerhans cell histiocytosis (LCH) is characterized by clonal neoplastic proliferation of bone morrow derived Langerhans cells in various tissues [1]. LCH is previously known as histiocytosis X, which includes a broad spectrum of clinical manifestations named eosinophilic granuloma, Hand-Schüller-Christian disease, and Letterer-Siwe disease, depending on the involved tissues [1]. The underlying etiology is obscure. LCH is a rare disease with an incidence of 1 : 200.000, and it is usually seen in children, less common in adults, and more common in men [2]. Focal lesions are generally diagnosed in older patients, particularly in postmenopausal woman. The most commonly involved tissues include bone, skin, lymph nodes, brain, and lungs. Female genital tract involvement is very rare, and vulva is the most common site being involved [3].

To the best of our knowledge, there are only 25 cases of pure vulvar LCH reported in the English literature, and approximately, only 7 cases are reported to be in the postmenopausal status [1, 4]. Therefore, it is hard to recognize and diagnose LCH of the vulva both for the clinician and the pathologist. For this reason, we want to call attention to the diagnosis of LCH by reporting a case of pure vulvar LCH in a postmenopausal woman. 

## 2. Case Presentation

**Figure 1:**
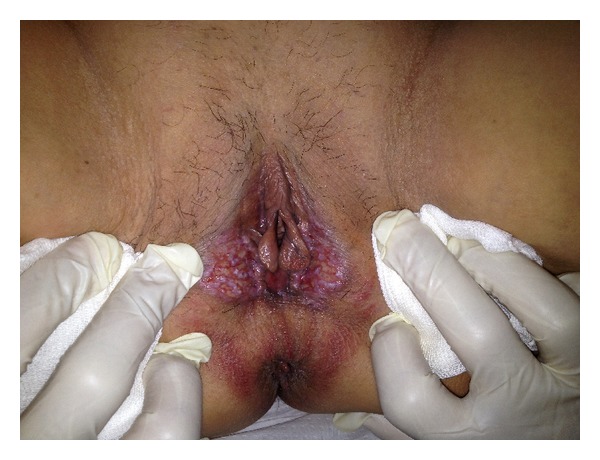
Vulvar lesion of Langerhans cell histiocytosis.

A 60-year-old woman was referred to our gynecology unit with vulvar itching, burning, and puffy skin lesions for 2 months. On physical examination, both labia majora and posterior fourchette ulcerated, and papillomatous lesions were detected without existing inguinal lymphadenopathy (Figure 1). She was gravida 0, and she had type 2 diabetes mellitus, and also, she was in postmenopausal state for 10 years. She has been taking oral antidiabetic agent gliclazide once daily. She did not use any hormone replacement therapy. The lesions were biopsied, and pathological diagnosis was LCH.

**Figure 2:**
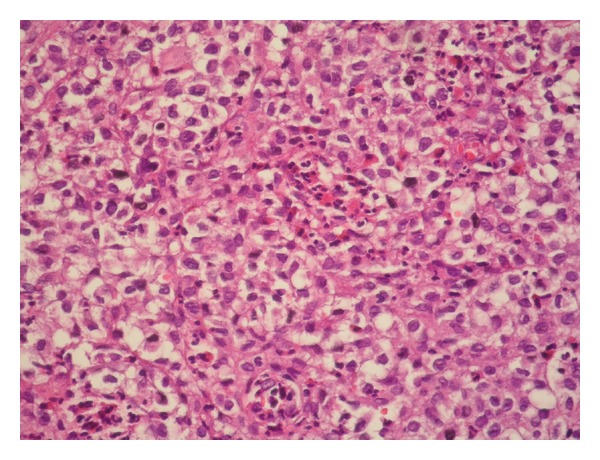
Neoplastic proliferation of Langerhans cells (hematoxylin and eosin staining ×200).

**Figure 3:**
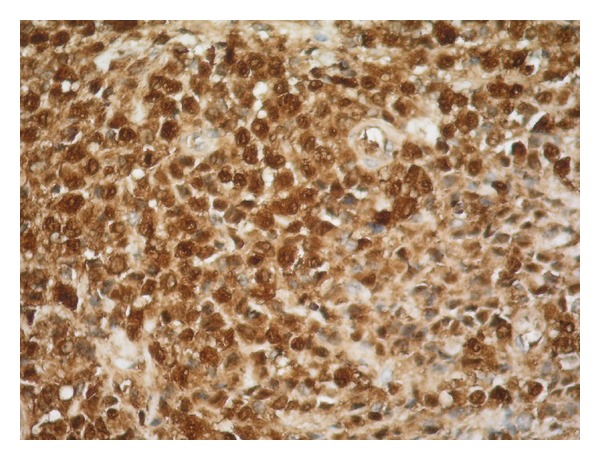
Langerhans cells staining positive for S-100 on immunohistochemical staining ×200.

**Figure 4:**
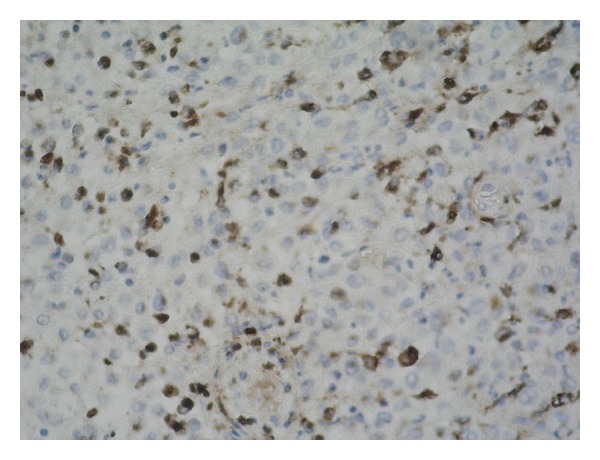
Langerhans cells staining positive for CD68 on immunohistochemical staining ×200.

Microscopic findings revealed ulcerated, keratinized stratified squamous epithelium and, under this, neoplastic cell infiltration both in the superficial and deep dermis. These uniform cells are ovoid in shape, with lobulated nucleus, and have large eosinophilic cytoplasm and ambiguous nucleoli (Figure 2). Immunohistochemistry showed strong positivity for S-100 (Figure 3), vimentin, CD1a, and CD68 (Figure 4) and weak positivity for Ki-67 in these cells.

To exclude systemic metastasis, high-resolution computed tomography of thorax and positron emission tomography scans were performed. These investigations revealed no metastatic disease except a vulvar hypermetabolic area 2 cm in the long axis that was diagnosed on PET scan. Additionally, a Pap smear test of the cervix and endometrial sampling was performed. Pathological reports of the smear test and endometrial sampling were appropriate with postmenopausal status. Tumor markers were also tested and found within normal limits.

The patient was informed about the treatment options, and she was offered surgical treatment (vulvectomy or local excision) combined with radiotherapy according to the Gynecologic Oncology Council opinion.

## 3. Discussion

In our case, we reported a very rare pure vulvar LCH in a postmenopausal woman. This is the 26th pure vulvar LCH case in the literature and the 8th pure vulvar LCH case in a postmenopausal woman [1, 4]. The diagnosis of vulvar lesions in the postmenopausal state is usually very challenging for the gynecologist. LCH usually presents as erythematous red plaques, eczematous, ulcerative, or polypoid lesions, which can easily lead to a misdiagnosis or interfere with other common vulvar skin lesions of postmenopausal period like lichen sclerosis, lichen planus, vulvar intraepithelial neoplasia, squamous cell carcinoma, candidiasis, psoriasis, contact dermatitis, Paget's disease, herpes simplex or human papilloma virus infections, and melanoma [5].

To distinguish between many vulvar diseases and to make the correct diagnosis, suggesting vulvar biopsy to the patient is the appropriate option. Pathological diagnosis is the gold standard for LCH. First, the key step for LCH diagnosis is the detection of neoplastic Langerhans cells with characteristic lobulated nuclei within eosinophilic leukocytes infiltrating the dermis on hematoxylin and eosin stain. Second, a positive immunohistochemical staining for CD1a, S-100, or CD68 is necessary and sufficient for a definitive diagnosis. Additionally, presence of Birbeck granules of neoplastic Langerhans cells on electron microscopy may help to improve the diagnosis [2, 6].

It is important to make the diagnosis of pure vulvar LCH in a postmenopausal woman, because many of the vulvar lesions in the postmenopausal period are usually treated with topical corticosteroids. In some cases, topical steroids may treat LCH, but, in some other cases, LCH may stay in remission during the treatment period, and after the cessation of therapy life-threatening distant metastases may occur if not checked [7].

There is still no universally accepted treatment protocol available both for vulvar and systemic LCH. In the reported cases of vulvar LCH, vulvectomy, local excision, radiotherapy, chemotherapy, and topical and oral steroids were used as treatment options. Among them, complete surgical excision is the recommended treatment. Radiotherapy is also beneficial in new cases. Spontaneous remission may also occur in some cases [1–4, 6, 7]. 

Although very rare, in a postmenopausal woman with symptoms like vulvar itching, burning, and ulcerated and papillomatous lesions, vulvar LCH should be considered in differential diagnosis, and definitive diagnosis should include immunohistological examination of biopsy material. Once the diagnosis is confirmed, distant organ metastases should be checked by radiology, and appropriate treatment options should be chosen.
